# Light-emitting diodes enhanced by localized surface plasmon resonance

**DOI:** 10.1186/1556-276X-6-199

**Published:** 2011-03-08

**Authors:** Xuefeng Gu, Teng Qiu, Wenjun Zhang, Paul K Chu

**Affiliations:** 1 Department of Physics, Southeast University, Nanjing 211189, People's Republic of China; 2 Current address: Chien-Shiung Wu College, Southeast University, Nanjing 211189, People’s Republic of China; 3 Department of Physics and Materials Science, City University of Hong Kong, Tat Chee Avenue, Kowloon, Hong Kong, People's Republic of China

## Abstract

Light-emitting diodes [LEDs] are of particular interest recently as their performance is approaching fluorescent/incandescent tubes. Moreover, their energy-saving property is attracting many researchers because of the huge energy crisis we are facing. Among all methods intending to enhance the efficiency and intensity of a conventional LED, localized surface plasmon resonance is a promising way. The mechanism is based on the energy coupling effect between the emitted photons from the semiconductor and metallic nanoparticles fabricated by nanotechnology. In this review, we describe the mechanism of this coupling effect and summarize the common fabrication techniques. The prospect, including the potential to replace fluorescent/incandescent lighting devices as well as applications to flat panel displays and optoelectronics, and future challenges with regard to the design of metallic nanostructures and fabrication techniques are discussed.

## Introduction

Light-emitting diodes [LEDs] have attracted much scientific and commercial interest since the realization of a practical LED device with emission frequencies in the visible region of the electromagnetic spectrum [1]. Since then, research activities have been focusing on how to produce economical LEDs with the desired colors as well as white light sources [2]. The strong demand has also driven materials technology, and new emitting materials and configurations have been proposed to enhance the performance. For example, the use of a polymer instead of small molecules opens the door to flexible, large-area, and stable organic LEDs [OLEDs] **[**3]. In the past 15 years, low-dimensional emitting devices incorporating quantum dots [QDs] and quantum wells [QWs] have been extensively investigated in order to achieve the desirable emission color and enhance device efficiency [4-10]. However, LEDs suffer from inherently low efficiency due to the sometimes low internal quantum efficiency [IQE] and difficulty extracting the generated photons out of the device. Although the use of electro-phosphorescent materials with proper management of both singlet and triplet excitons has brought IQE in OLEDs to almost unity [11-13], that of LEDs with inorganic emitting materials such as GaN, CdSe, and Si QDs or QWs remains unsatisfactory because non-radiative electron/hole pair recombination dominates. Another channel of energy loss is total internal reflection at the emitter/air interface because of the typically high refractive index of the emitting materials. Several methods have been proposed to enhance the overall efficiency of LEDs, and they include substrate modification and incorporation of scattering medium, micro-lenses, nanogratings, corrugated microstructures, photonic crystals, and so on [14-17]. In spite of some efficiency enhancement, spectral changes and angle-dependent colors associated with the substrate modification techniques, the high precision needed to produce nanogratings and the high cost of photonic crystals are still challenging issues plaguing commercial applications.

**Figure 1:**
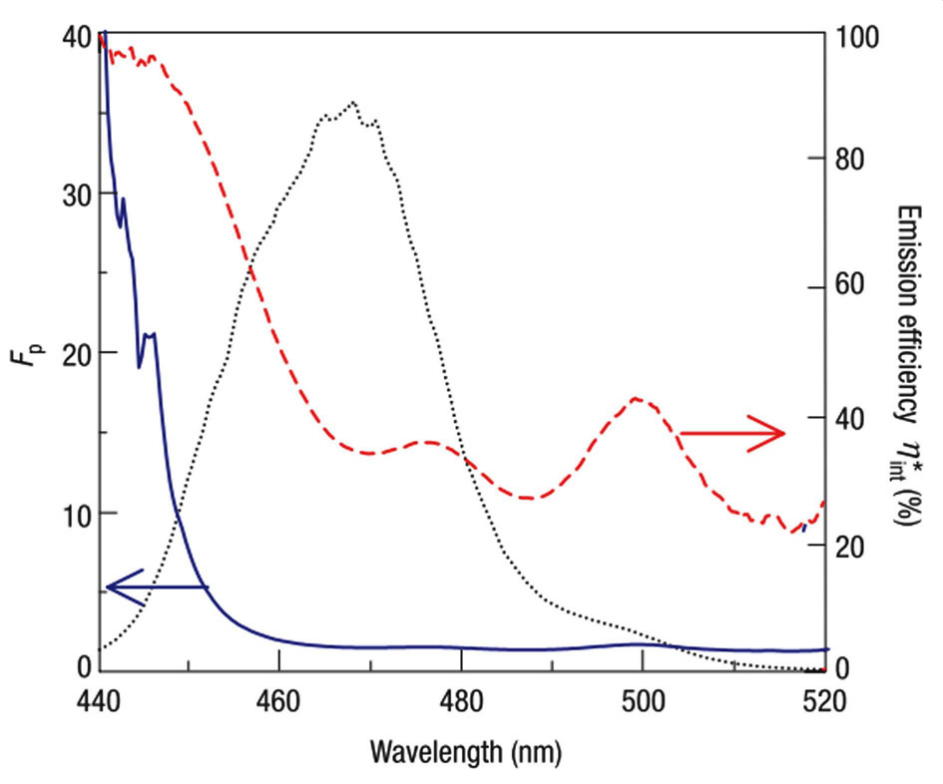
**Enhanced emission efficiency, Purcell factor, and PL spectrum of the sample**. These are shown as *red dashed line*, *blue solid line*, and *black dotted line*, respectively. Nearly 100% emission efficiency can be obtained at around 440 nm; however, this does not perfectly match the emission peak. Reproduced from [18]. Copyright Nature Publishing Group, 2004.

Surface plasmon polaritons [SPPs] were first exploited to enhance the efficiency of InGaN QW-based LEDs by Okamoto et al. in 2004 [18]. Known as Purcell effect, when the resonant frequency of the silver SPPs overlaps the emission frequency of the InGaN QWs, the energy coupled to the SPP mode is significantly increased and thus the IQE is enhanced [19]. Scattered by the rough silver film, the energy coupled to the SPP mode can be recovered as free space photons. In their work, the enhanced IQE η*_int _is observed to increase 6.8 times with Ag coating, leading to a very desirable Purcell factor(1)

where *η*_int _represents the original IQE. Figure 1 shows the wavelength-dependent η*_int_, Purcell factor, and the emission spectrum of their sample. It is clear that greater enhancement can be obtained at shorter wavelengths (~440 nm). However, this wavelength does not perfectly overlap the GaN/InGaN emission peak, leaving space for better enhancement. In fact, the SPP resonant energy must be in the vicinity of the emission energy in order to achieve the best enhancement. This rule has since been verified by other experiments [20-23]. Hence, only a small subset of LEDs can be enhanced via SPP/emitter coupling because the SPP resonant frequency of a metal film cannot be easily tuned. Another challenge is that the metal film is typically opaque, thereby making light extraction from the metal side of the device difficult. It has been shown that light can be effectively extracted from the metal side by exploiting the surface plasmon cross-coupling effect, but incorporation of the appropriately scaled nanostructures is necessary [24,25].

**Figure 2:**
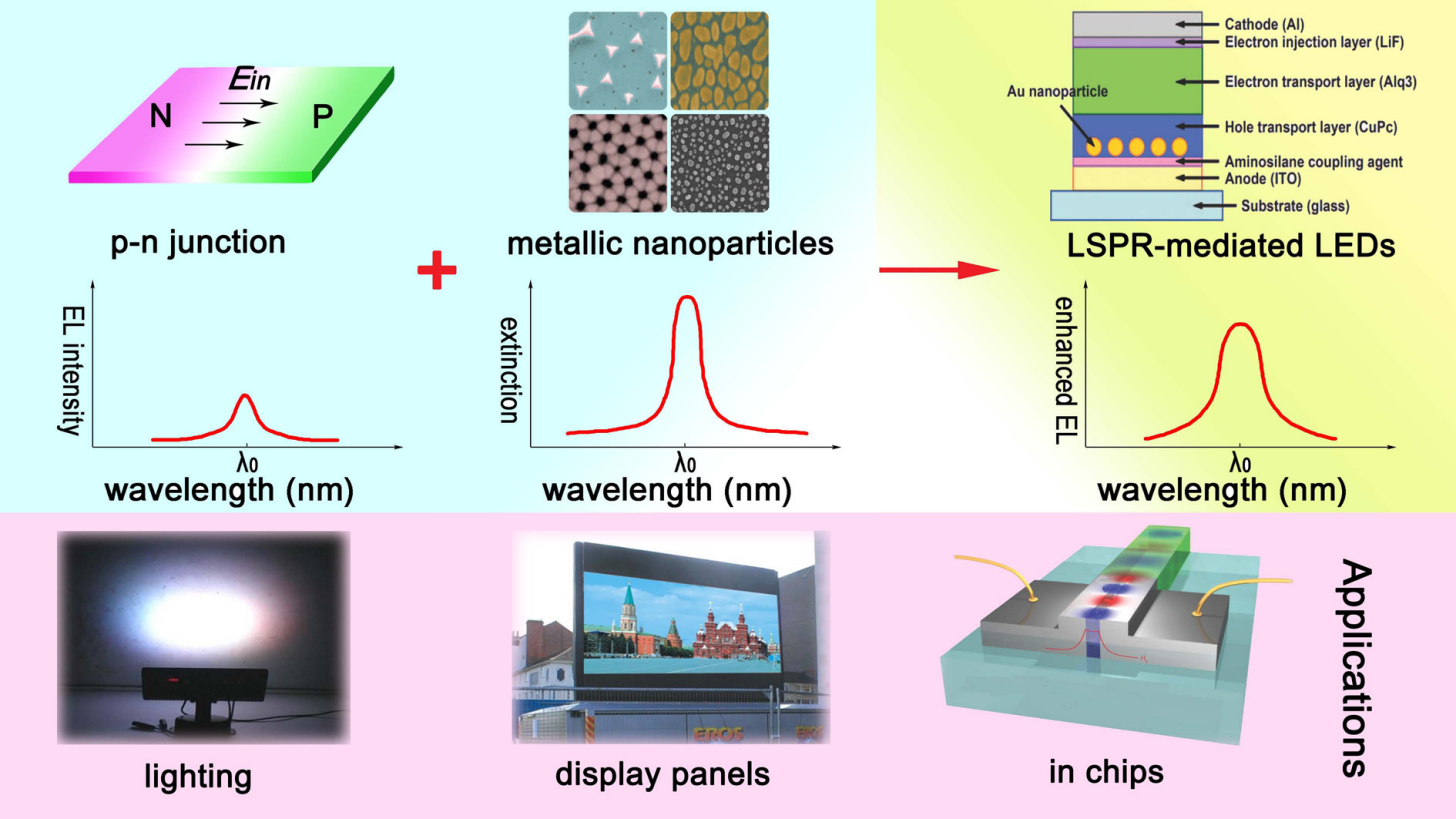
**Noble metallic NP layer deposited on or within a conventional LED to enhance efficiency of device**. This new class of LEDs can be used in various compelling applications.

In comparison with the aforementioned technology, localized surface plasmon [LSP] offers a unique advantage in tunability; that is, the optical properties resulting from LSP can be easily varied by altering the type, size, geometry, and interparticle distance of the metallic nanoparticles [NPs]. The other advantage of LSP-enhanced LEDs over SPP ones is less dissipation since the induced wave is locally confined and cannot propagate along the metal surface. Furthermore, the metal layer is no longer opaque, making emission from the metal side possible, and so metallic NPs instead of a continuous metal film can be used to enhance the performance of LEDs. Figure 2 schematically shows the story of this review: incorporation of noble metallic nanoparticles into LEDs leads to a new class of highly efficient solid-state light sources (top row); in order to get considerable enhancement, the extinction band of LSP must be close to the band-gap emission energy of the LED (middle row); and this new technology has found its applications in general lighting, flat panel displays, and ultrafast optoelectronic chips (bottom row). Recent improvements combined with the low cost and easy fabrication process make localized surface plasmon resonance [LSPR]-enhanced LEDs very attractive commercially.

## Mechanism

Pioneering experimental results have confirmed the importance of overlapping between the LSPR energy and emission energy, and some of them are presented in Table 1. The type, shape, height, and density of the NPs determine the degree of enhancement. In order to explore the cause and mechanism, experiments have been conducted carefully, often excluding other possible factors which may contribute to the enhancement such as reflection from the metallic NPs, emission from the NPs themselves, increased absorption of light in photoluminescence [PL] enhancement, and quenching of defects emission. However, although it is generally agreed to stem from resonant coupling between the semiconductor band-gap emission and LSP generated by the metallic NPs, the exact mechanism is still debatable. In this section, we discuss two mechanisms that have been suggested to explain the resonant-coupling enhancing effect, namely, increase of IQE via emitter/plasmon coupling and increase of light extraction efficiency [LEE] by means of out-coupling of the generated photons.

**Table 1:** Summary of representative experimental results showing the important relationship between the LSPR energy and emission energy in order to attain the best enhancement

Emitting materials and configurations	Peak emission energy (nm)	Metal used	Optical properties of metal layer	Enhancement	References
InGaN/GaN multiple QW	463	Ag	Transmittance exhibits absorption from 396 to 455 nm	32.2% with an 100-mA current	Kwon et al. [33]
Alq_3 _thin film	525	Au	A peak in absorption at 510 nm	20-fold	Fujiki et al. [34]
InGaN/GaN QW	550	Ag	A dip in transmission at about 550 nm	150% in peak intensity with a 20-mA current	Yeh et al. [36]
InGaN/GaN QW	465	Au	A dip in transmission in 511 nm	180% with a 20-mA current	Sung et al. [37]
Organic poly	575	Ag	Large absorption from 330 to 500 nm	Sixfold	Qiu et al. [41]
Si QD	600	Ag	A peak in absorption at 535 nm	Reaches maximum at 530 nm	Kim et al. [42]
ZnMgO alloys	357	Pt	Extinction band near 350 nm	Sixfold	You et al. [44]
ZnO film	380	Ag	Extinction band near 370 nm	Threefold	Cheng et al. [45]
Si-on-insulator	1,140	Ag	A dip in transmission at about 520 nm	2.5-fold in peak intensity	Pillai et al. [48]
CdSe/ZnS nanocrystals	580	Au	A peak in absorption at about 600 nm	30-fold	Pompa et al. [53]
Si QD	775	Ag	A dip in transmission at 710 nm	Twofold, with the peak blue shifts	Biteen et al. [54]
GaN	440	Ag	A dip in transmission at about 440 nm	Twofold	Mak et al. [62]

### Enhancement of IQE

The local electric field and magnitude of the extinction spectrum are significantly enhanced at the LSPR frequency [26]. This effect has been broadly studied and utilized in many fields such as surface-enhanced Raman spectroscopy, solar cells, and biosensors [27-30]. With regard to the efficiency improvement rendered by LSPR, it is supposed that the enhanced electric field interacts with the emitting materials, increasing the spontaneous emission rate and consequently enhancing the IQE of the device. This assumption can be partly verified by experiments showing that the radiative decay rate and spontaneous emission rate of the light emitters can be improved in the presence of silver SPPs since the enhanced local electric field at the LSPR frequency plays a similar role as the evanescent wave induced by SPPs [31,32]. An example of the IQE enhancement in LSPR-enhanced LEDs is the GaN-based LED developed by Kown et al. [33]. The optical output power increases by 32.2% at an input current of 100 mA, and the time-resolved PL measurement shows that the PL decay time in the presence of Ag NPs is significantly reduced. As a result, the spontaneous emission rate and the IQE are better.

The effect of varying the distance between the active emitting region and metallic NPs on the overall device efficiency enhancement also reveals that the larger local field plays an important role. Coupling and enhancing vanish as the distance is increased above a certain threshold, and the distance cannot be too small or too large. If it is too small, the non-radiative quenching process dominates and most of the energy is dissipated accordingly. On the contrary, if the distance is too large, the coupling effect vanishes since only the electron/hole pairs near the metallic NPs can effectively couple to increase the IQE. Fujiki et al. [34] have introduced a copper phthalocyanine hole transport layer in their LED structure as a spacer to avoid non-radiative quenching. In order to retain the effect, a 20-nm-thick film is used and smaller enhancement is observed if the thickness is larger. The experiment provides direct evidence of the role played by the higher local electric field in the IQE enhancement effect.

**Figure 3:**
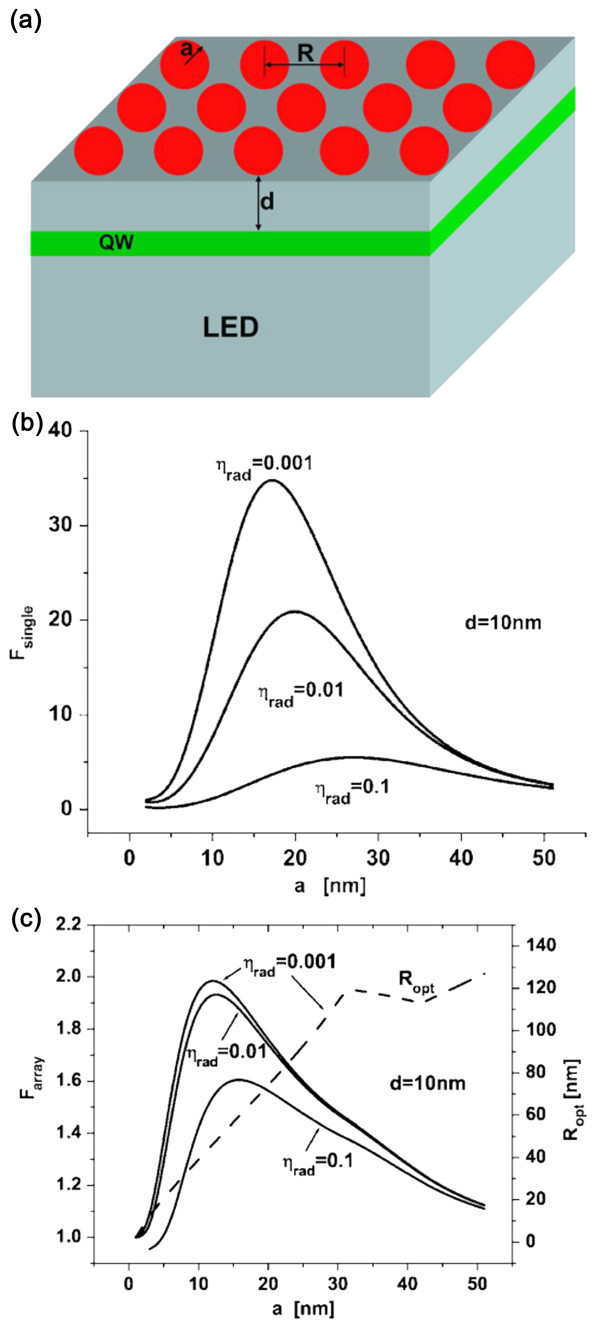
**Use of NPs to enhance the IQE of LEDs**. (**a**) Two-dimensional ordered array of metal NPs placed in the vicinity of the QW active region of a LED. (**b**) Enhancement due to isolated Ag spheres on InGaN/GaN QW emitters with a separation of 10 nm as a function of the sphere radius *a *for different original radiative efficiencies. (**c**) Enhancement due to two-dimensional array of Ag spheres on InGaN/GaN QW emitters with a separation of 10 nm as a function of the sphere radius *a *for different original radiative efficiencies. Also shown is the optimized sphere spacing *R*_opt _for *η*_rad _= 0.001. Reproduced from [35]. Copyright American Institute of Physics, 2008.

A model proposed by Khurgin et al. [35] further confirms the importance of the higher local field and demonstrates that the IQE can indeed be enhanced by LSPR via the electric field/emitter interaction. However, the use of NPs to enhance the IQE of LEDs is effective only when the original IQE is very low (<1%) and the NPs are highly disordered, as shown in Figure 3. The results are corroborated by experiments. For example, Yeh et al. [36] have studied two InGaN/GaN QW-based LEDs emitting different colors and discovered that the green one, which has a lower original IQE corresponding to a lower crystal quality, exhibits a more effective enhancement than the blue one. Besides, due to indirect band-gap emission, Si has a relatively low original IQE and can possibly gain the most from LSP, which will be discussed in "Applications" section.

### Enhancement of LEE

**Figure 4:**
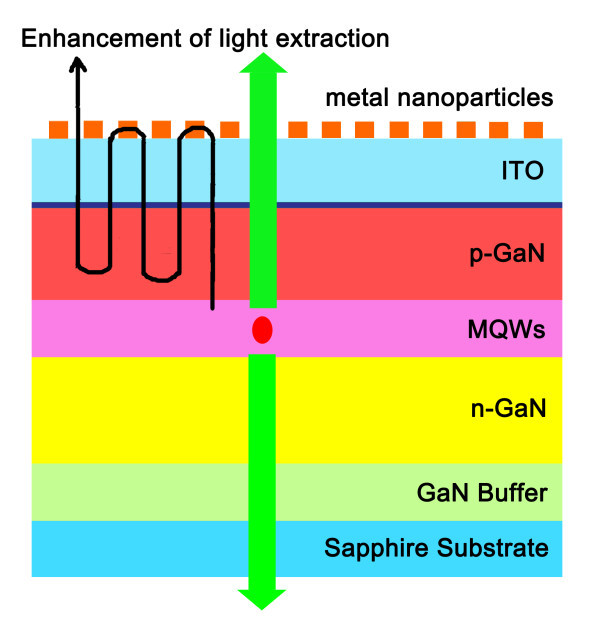
**LEE enhancement of a GaN-based LED**. Schematic illustration of the structure of an electroluminescent LED in which LEE is enhanced via energy transfer.

In some situations, LSPR is expected to enhance the LEE rather than IQE. In this case, the energy of the generated photons is first transferred to the metallic NPs to induce LSP followed by emission of light. While IQE enhancement has been studied, only a few cases in which the LEE is enhanced by LSP have been reported. The GaN-based LED developed by Sung et al. [37] and described in Figure 4 shows an electroluminescence [EL] increase of 1.8 times at an injection current of 20 mA. As the distance between the gold NPs and the GaN multiple QW [MQW] is very large, the argument about enhancement by emitter/field interaction no longer holds, and so the enhancement can only stem from an out-coupling of the generated photons by the gold NPs.

From the viewpoint of energy transfer, nanoscaled antennae can be incorporated to enhance the absorption of generated photons and subsequent reemission. As the boundary separating the fields of electronics and photonics is becoming more blurred, the concept of antennae developed for radio frequency and microwave communication has been extended to optical frequencies, and one potential application of these nanoscaled antennae is to enhance light extraction from light emitters [38,39]. In the paper by Bakker et al. [40], a nanoantenna system consisting of two gold elliptical NPs is shown to enhance the LEE of a fluorescent dye by a factor of 20 to 100. Near-field measurements show that the enhanced emission is localized and polarized. Another group has reported an organic emitter coupled with silver nanoantenna arrays [41]. In addition to increased light absorption in the ultraviolet [UV] range and enhanced PL efficiency, an energy transfer process responsible for light extraction is suggested. In fact, the effect of increased UV absorption is less dominant compared to the enhancement of LEE, as reported in similar experiments, and the results demonstrate the efficacy of nanoantennae in efficiently extracting generated photons.

## Fabrication

Vacuum deposition such as sputtering or electron beam evaporation sometimes accompanied by post thermal processing [PTP], electron beam lithography [EBL], nanosphere lithography [NSL], nanoimprinting, and chemical synthesis are common methods to produce metallic NP arrays. However, not all of these techniques have been used to make LEDs due to various technological considerations. For instance, many of these methods involve chemical processing, which can harm the LED structure. At present, vacuum deposition is the most successful in fabricating EL LEDs, whereas only PL results have been obtained from samples produced by other techniques. Nonetheless, the PL results still have considerable value as they enable better understanding of the nature of the LSPR-induced enhancement and spur further development in the fabrication methods. In this section, we not only describe these various methods but also discuss the compatibility of each method from the perspective of LED fabrication, ease of implementation, and production cost.

### Vacuum deposition

Under ultrahigh vacuum conditions, a thin metal film can be formed on the LED structure via the condensation of atoms produced by evaporation. The film thickness is governed by factors such as the substrate type, ambient pressure, and sputtering time. One advantage of the technique is that it does not require chemical processing, and so chemical damage to the emitting region can be avoided. This method has been employed to produce Ag NP arrays on a silicon QD LED [42]. To alter the optical properties such as the extinction spectrum of the materials for better enhancement, PTP is often applied. By conducting annealing at different temperatures and for different time durations on metal films with different thicknesses, NPs with the desirable sizes and heights can be produced in order to achieve the best coupling effect with the light emitters. Yeh et al. [43] have observed that the size and heights of Ag NPs are significantly increased after annealing three samples with initial film thicknesses of 5, 10, and 15 nm at 200°C for 30 min. Annealing enhances the PL intensity of the LSPR light emitters due to enhanced LSP/QW coupling. Other PL experiments conducted on Pt/ZnMgO films [44], Ag/ZnO films [45], and conjugated polymers [46] also demonstrate the constructive role of annealing after vacuum deposition in enhancing the coupling between the emitters and metallic NPs.

**Figure 5:**
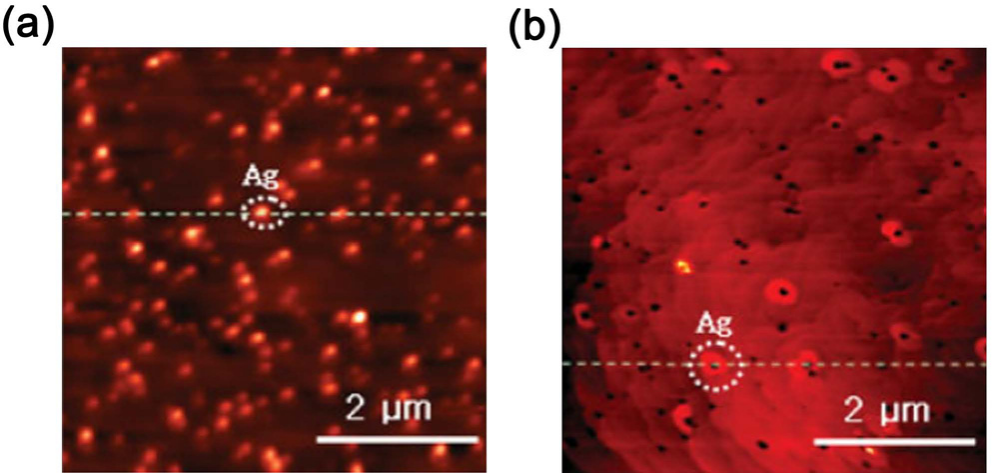
**AFM images of Ag NPs deposited on an n-GaN layer before and after annealing**. (**a**) Before annealing and (**b**) after annealing. After deposition, the particle size is significantly enlarged for better enhancement. AFM, atomic force microscopy. Reproduced from [33]. Copyright WILEY-VCH Verlag GmbH & Co. KGaA, Weinheim, 2008.

Owing to the ease of implementation and reasonable cost, vacuum deposition together with PTP is the most common method in fabricating LEDs containing metallic NPs. Figure 5 shows a typical image of Ag NPs deposited on an n-GaN layer before and after annealing [33]. Similar to the results described in [43], after annealing at 750°C for 10 min in a metal/organic chemical vapor deposition chamber, the size and height of the NPs change from 275 ± 50 and 8 ± 4 nm to 450 ± 50 and 15 ± 5 nm, respectively. Afterwards, a 20-nm-thick undoped GaN, 22-nm-thick InGaN/GaN MQW, and a 0.2-μm p-type GaN layer are deposited onto the Ag NPs and two electrodes are added to the LED structure. The output optical power is observed to increase by 32.2% at an input current of 100 mA due to the enhancement of IQE resulting from coupling between the MQW and Ag NPs. In addition, InGaN/GaN MQW LEDs with different structures [36,37,47] and Si-based LEDs [48] have been produced by the sputtering-annealing process.

### Electron beam lithography

**Figure 6:**
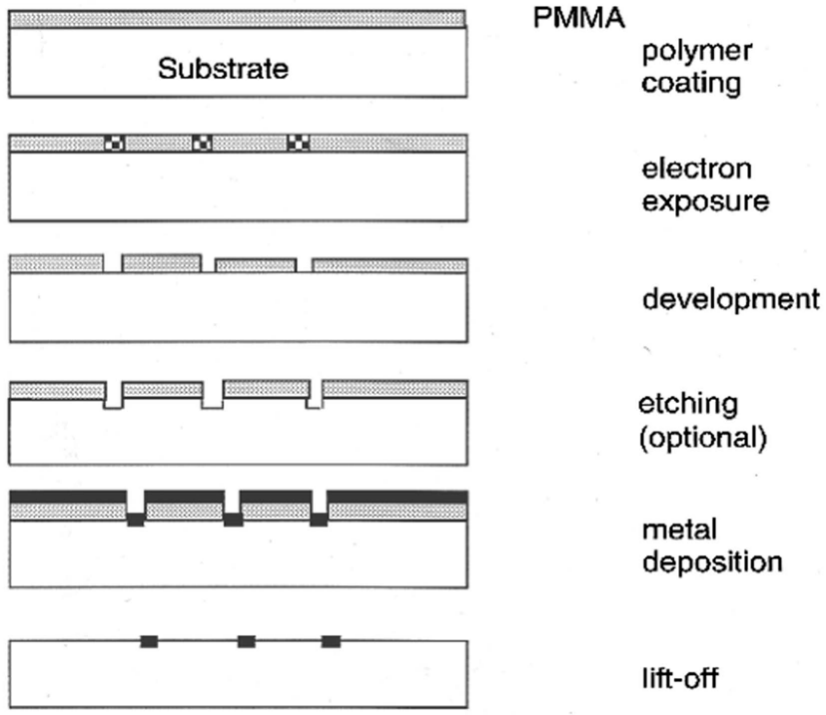
**General procedure of EBL**. Reproduced from [50]. Copyright American Chemical Society, 1997.

**Figure 7:**
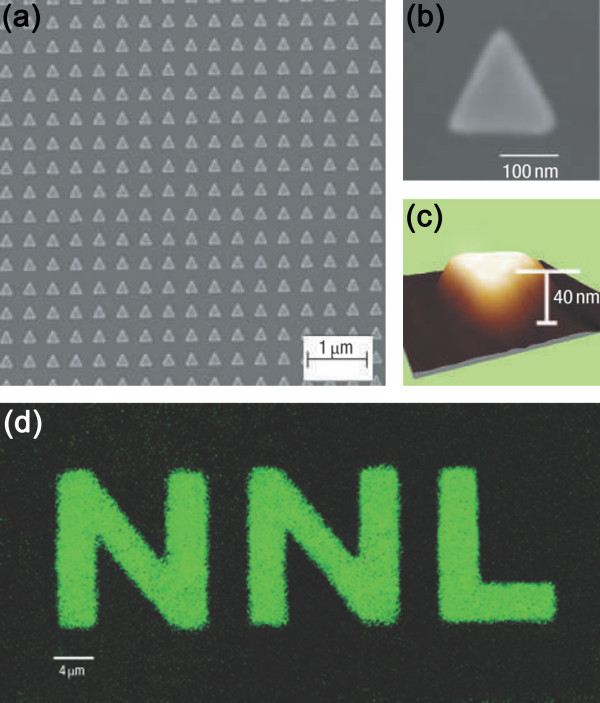
**Typical example of a highly regular gold nanopattern by EBL**: (**a**) SEM image of a triangular gold array. (**b**) High-resolution SEM image of a single nanotriangle. (**c**) AFM image of the single nanostructure. (**d**) Comparison between fluorescence with and without Au NPs. SEM, scanning electron microscopy. Reproduced from [53]. Copyright Nature Publishing Group, 2006.

EBL is often used to fabricate sub-100-nm monodispersed metallic NPs. Current state-of-the-art equipment boasts a resolution of < 10 nm that is small enough for LSPR-enhanced LEDs. The general procedures to produce metallic NPs are illustrated in Figure 6, and more details can be found elsewhere [49-52]. EBL can be utilized to produce metallic NPs exhibiting enhanced light emission efficiency. For example, metal-enhanced fluorescence has been reported by Pompa et al. [53]. Here, EBL is used to fabricate highly ordered 100- to 200-nm-wide triangular gold prisms on planar substrates, and pictures of one representative sample are depicted in Figures 7a,b,c. Uniform CdSe/ZnS core/shell nanocrystals [NCs] dispersed in a polymethylmethacrylate [PMMA] matrix to control the distance between the NCs and gold NPs are spin-coated onto the substrate, and the resulting enhancement is as high as 30-fold, as demonstrated by a comparison of the fluorescence with and without Au NPs in Figure 7d. Besides CdSe/ZnS NCs, luminescence from Si QDs increases by a factor of 7 [54]. After cleaning the Si QD-doped quartz in a 5:1:1 H_2_O/H_2_O_2_/NH_4_OH solution, EBL is used to pattern 100 × 100-μm circular arrays on two layers of PMMA covered by Ge. After removing the Ge and developing the resist, the Si and Ag layers are deposited by vacuum deposition, and following lift-off of the remaining PMMA, only Ag islands are left on the quartz.

Although EBL has some benefits over other fabrication methods, for instance, the high resolution and the ability to produce NPs with different shapes, it has disadvantages. For instance, large-area fabrication is difficult. The NPs produced are only two-dimensional and the equipment is expensive. Another disadvantage is that the acetone lift-off process harms the electron or hole transport layer. Future research needs to focus on introducing some protection during patterning of the metallic NPs.

### Nanosphere lithography

**Figure 8:**
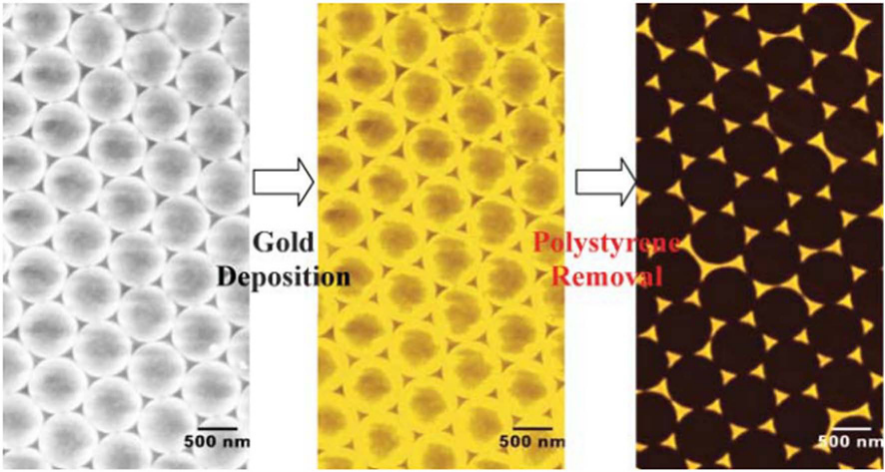
**Illustration of the NSL process**. Shown are the deposition of polystyrene spheres on the substrate, thermal evaporation of gold, and removal of polystyrene spheres leaving triangular gold NPs. Reproduced from [52]. Copyright The Royal Society of Chemistry, 2006.

Traditional NSL, as illustrated in Figure 8, involves dropping a suspension of nanospheres onto a substrate, self-assembly into a hexagonally closely packed two-dimensional colloidal crystal that serves as a deposition mask, vacuum/electron beam deposition, and finally, removal of the nanosphere mask by ultrasonic treatment in an organic solution [55-57]. Different from EBL, the patterning is based on self-assembly rather than electron beam irradiation. Hence, the production cost is reduced because only a small amount of nanosphere solution is needed. In addition, the use of copper instead of Au, Ag, and Pt without degrading the NP properties can lead to further low production cost [58]. Other NSL-based techniques, for example, double-layer mask-based and angle-resolved NSL, allow more flexible tuning of the metallic NP optical response [59,60] and, consequently, enhancement in light emission. NSL has been utilized to fabricate metallic NPs to increase PL from Si nanocrystals and GaN light emitters [61,62]. However, on account of inherent restrictions such as difficulty to fabricate structures other than triangles or quasi-triangles, this method has not been extended to produce LEDs.

### Using templates

Using patterned templates, various nanostructures, both two- and three-dimensional, for example cylinders, squares grooves, and pyramids, can be easily fabricated. Frequently used templates are often produced from porous anodic alumina [PAA] or by lithography techniques [41,63,64]. The advantage of this method is avoidance of chemical peel-off, thus making incorporation of metallic NPs into EL LEDs possible. At present, only PAA-based templates have been applied to the fabrication of PL light emitters [41], and the bright future of using templates to fabricate EL LEDs is to be explored.

### Other methods

The fabrication techniques aforementioned are mainstream techniques that have been applied to NP fabrication to improve light emission efficiency. Nanoimprint lithography and chemical synthesis are alternatives and have been extensively studied [65-67]. However, these two techniques are too costly or difficult to control, and so neither of them has been used successfully to produce NP arrays with improved light emission. More work is required in order to produce large-area, cost-effective, and easily tunable metallic NPs with enhanced light emission efficiency. One promising proposal for achieving this goal is to use self-assembled nanorods, nanowires, or nanotubes because of the ease of production and high controllability [68-71]. Ag nanorods formed by heating of AgNO_3 _in pores of PAA template or oblique angle deposition, nanotubes by shadow evaporation, and Ag NPs on stacked carbon nanotube layers have already found intriguing applications in surface-enhanced Raman scattering, and we anticipate that these proposals may shortly be employed to LED efficiency enhancement with proper modifications.

## Applications

LEDs have found major commercial applications in three areas: general lighting, flat panel displays, and optoelectronic chips.

### General lighting

**Figure 9:**
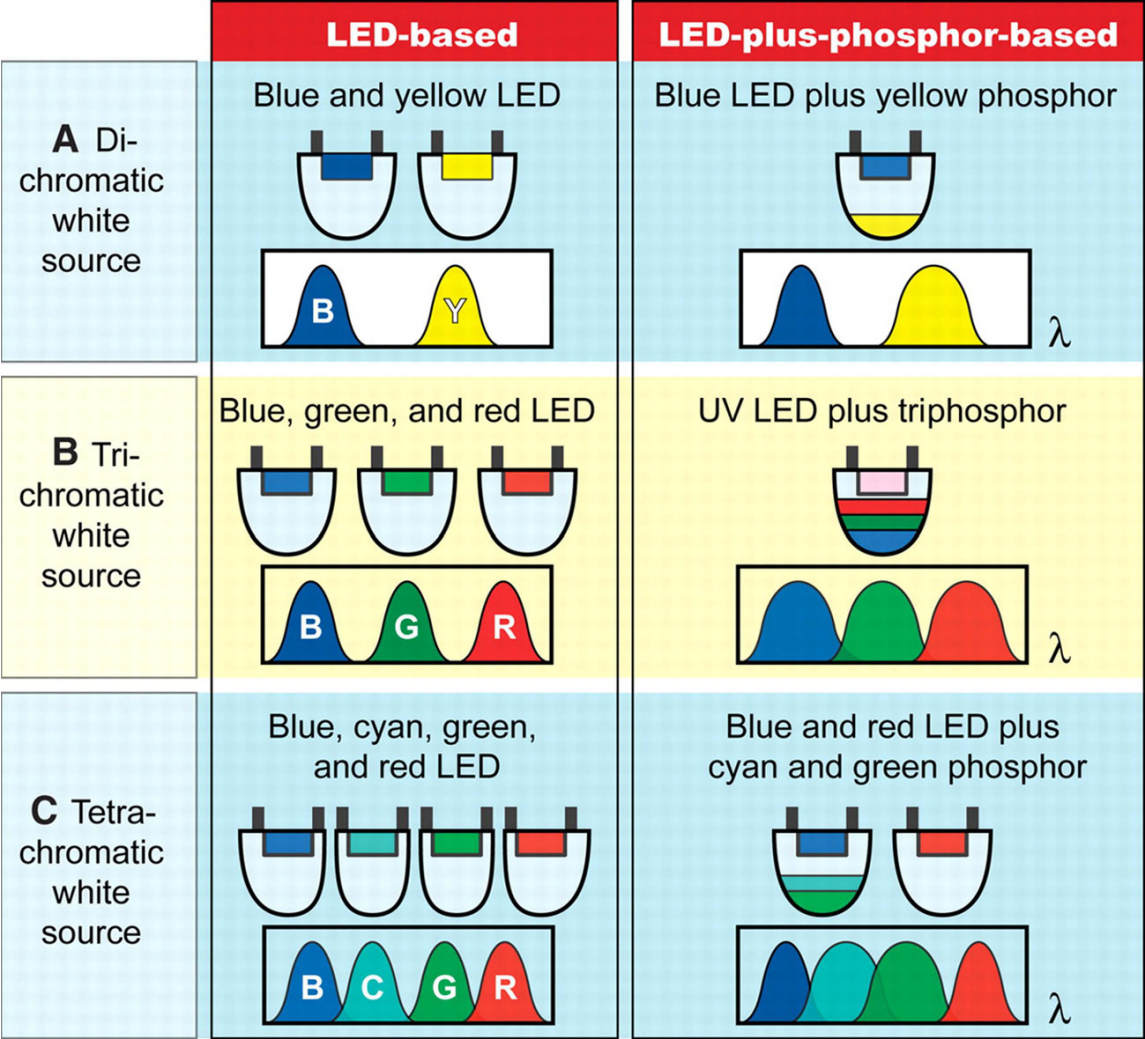
**LED-based and LED-plus-phosphor-based approaches for white light sources implemented as di-, tri-, and tetrachromatic sources**. The trichromatic approaches can provide a reasonable trade-off between color and luminous source efficiency. Reproduced from [72]. Copyright the American Association for the Advancement of Science, 2005.

Advantages such as energy saving, low radiation, shock resistance, and spectral power tunability over fluorescent and incandescent light sources have propelled the commercialization of solid-state lighting devices containing semiconductors as the light emitters [72]. Specifically, recent realization of LEDs with fluorescent tube efficiency makes the prospect of solid-state lighting brighter. Some common strategies to make white light LEDs are illustrated in Figure 9. One method is to combine emitters with different colors in the device and adjust the spectral composition according to practical needs. Alternatively, phosphors and certain semiconductor NCs such as CdSe/ZnS core/shell quantum dots are known to be capable of effectively converting high-energy photons to low-energy ones, and consequently, white light sources can be produced. In both strategies, the use of a blue or UV LED is indispensable. However, typical inorganic blue light emitters based on GaN/InGaN QWs usually have a low IQE due to the relatively poor crystal quality. Since LSPR can introduce efficiency enhancement when the original IQE is low, there is big interest in using LSPR to increase the overall efficiency of short-wavelength blue and green LEDs, as discussed in previous sections.

The use of LSPR in fabricating white LEDs has been described by Yeh et al. [73]. Here, mixing of the red light converted from blue/green photons and residual blue light leads to white light emission. Instead of enhancing the IQE or LEE of the device, this configuration enhances light absorption by the CdSe/ZnS QWs via coupling between the QWs and LSP generated by the Au NPs. Spectral tunability, high quantum efficiency, and photo-stability are the advantages of this method. Another possible but seldom reported method to increase the efficiency of white light emitters is to incorporate NPs of different geometries in a device. As demonstrated in microwave frequencies, combination of NPs with various geometries results in several transmission dips corresponding to the type of NPs [74]. Hence, if different NPs with properly designed geometries are introduced into a white light emitter, the various light components can be enhanced selectively, thereby producing light emitters giving the desirable spectrum.

### Flat panel displays

**Figure 10:**
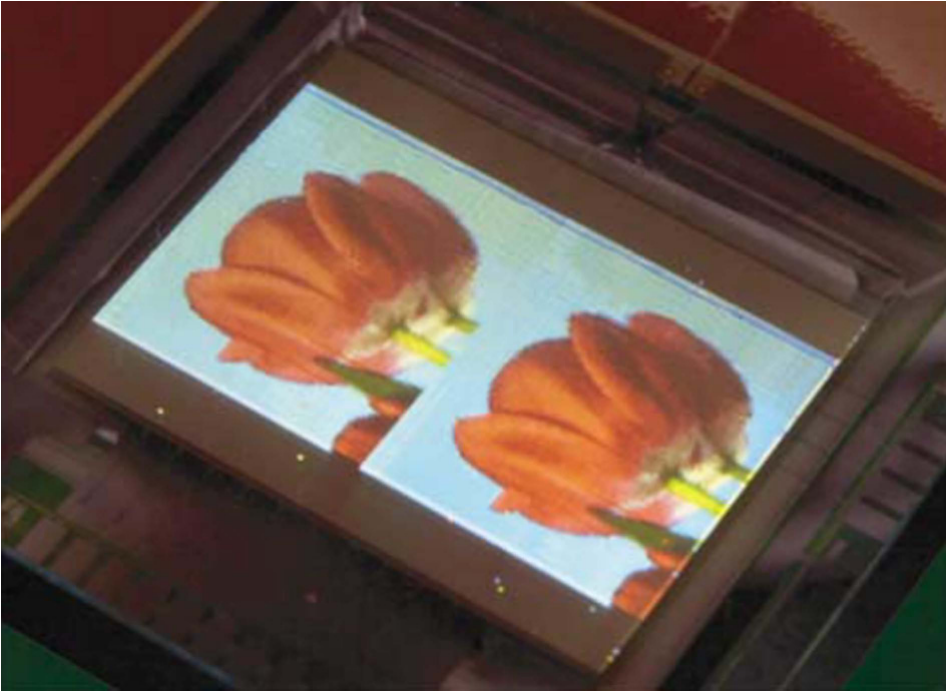
**Image of an organic display panel developed by Philips Electronics**. Reproduced from [75]. Copyright Nature Publishing Group, 2004.

Commercial flat panel displays are typically liquid crystal [LCD] and plasma displays [PD]. Although these two schemes dominate the market today, many problems still remain unsolved, for instance, narrow viewing angle in spite of big improvement since its inception, poor resistance to shock associated with LCDs, complexity in small-area fabrication and UV radiation inherent to PDs. Consequently, scientists continue to search for other display schemes and devices. LED is a viable alternative due to the high intensity and energy saving. Unlike LCDs, commercial LED displays usually adopt organic emitting materials, and an image of which is shown in Figure 10[75]. OLEDs have attracted considerable attention due to advantages such as flexibility, good intensity, and large area. Both indoor (televisions) and outdoor LED displays have been introduced to the market, and LSPR can be used to enhance the efficiency of these LEDs (mainly LEE). Meanwhile, the ability of performing light extraction from LEDs enables LSPR to be an effective top emitter (emission from the metal side instead of the substrate). In addition, utilization of LSPR obviates the need for carefully designed surface structures, thus reducing both the fabrication complexity and cost. Although a commercial display with LSPR-enhanced light extraction has not yet been made, there have been encouraging experimental results concerning the efficiency, color composition, and top emission capability, which are instrumental to the practical application of LSPR-enhanced LEDs.

### Optoelectronic chips

**Figure 11:**
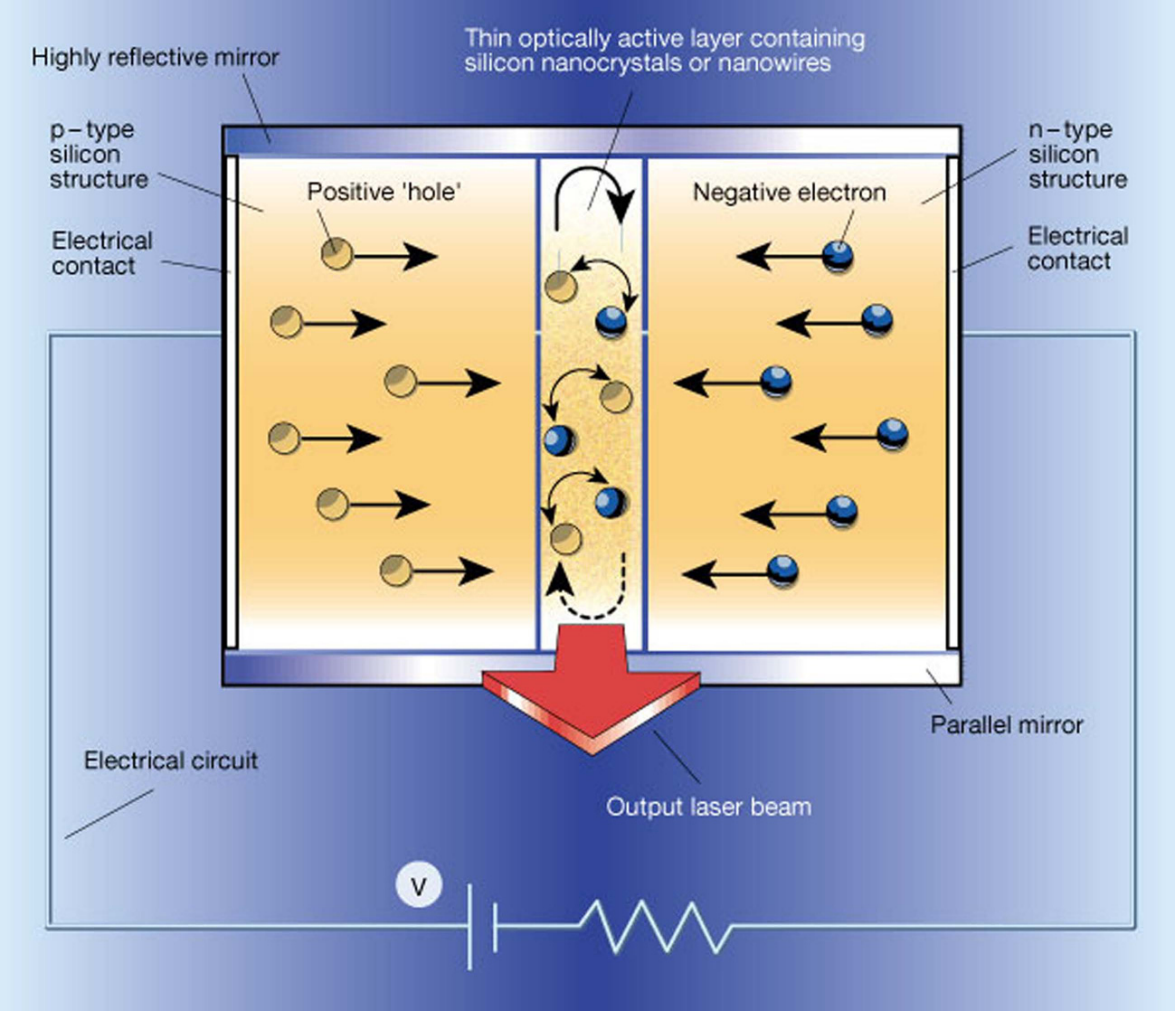
**A future electrically driven Si laser diode with two highly reflective mirrors**. The mirrors act as the optical cavity to amplify light emission. Reproduced from [79]. Copyright Nature Publishing Group, 2000.

Digital data transport in state-of-the-art microprocessors encompassing ultrafast transistors requires high-speed interconnects, and optical components like optical fibers can satisfy the needs. Despite a capacity 1,000 times larger than their electronic counterparts, these dielectric interconnects are limited in size by the fundamental laws of diffraction to the order of wavelengths. This means that the size must be at least one order of magnitude larger than the electronic elements, making it rather difficult to integrate electronic and photonic devices into the same optoelectronic chip [76,77]. Surface plasmon [SP] is among the most promising substitutes for optical fibers. Here, nanometer-scaled structures can serve as the SP waveguides, modulators, and switches in a communication system. Si-based laser diodes are promising light sources to generate SP in these optoelectronic chips because of the low cost, ease of integration into traditional integrated circuit technologies, and large-area production. However, as indirect band-gap materials, the IQE of Si emitters is very low. Methods to enhance the efficiency and to increase the visible components in the Si emission spectrum include the introduction of porous Si and quantum dots which have raised the IQE of Si-based light emitters to 1%. Since the report of high-efficiency visible PL from Si quantum dots in 2000 [78,79], the EL properties of Si QDs have been extensively investigated in an attempt to produce an all-silicon laser diode, as shown in Figure 11[80-82]. Kim et al. [42] have developed a LED structure with a Ag layer containing Ag NPs between the silicon nitride layer containing the Si QDs and Si substrate. This device yields an EL intensity that is 434% larger than that without NPs. The enhancement is attributed to the enhanced IQE, which is expected because the LSPR-induced enhancement is more substantial if the original IQE is relatively low. A Si-on-insulator LED with higher efficiency rendered by Ag NPs has been reported by Pillai et al. [48]. By adopting a simple deposition process, an eightfold efficiency increase at 900 nm stemming from LEE enhancement is achieved. Although commercial all-Si laser diodes have not yet been made, there is great potential and much research is being conducted in this area.

## Conclusion

Among the various techniques to improve the efficiency of LEDs, LSPR-based methods have great promise due to the high degree of enhancement and reasonable cost. The enhancement is generally attributed to the increase in the IQE or LEE of the device. By using traditional nanotechniques such as sputtering/deposition, EBL, and NSL, LEDs with a myriad of metallic NP geometries have been fabricated. By carefully examining their PL or EL properties, the enhancement mechanism has been elucidated, further confirming the immense potential of LSPR LEDs.

One of the current tasks is to identify the suitable fabrication parameters and optimize the size and shape of the structure in order to achieve the best enhancing effect experimentally. Analytical expressions have been obtained for the interaction between simple nanostructures like nanoholes and light [83]. More powerful are computer softwares, such as finite difference time domain [FDTD] tools, for it is flexible to simulate any structures with them, from regular to highly disordered. As a common practice in the emerging field of metamaterials such as sub-wavelength metallic structures, it is routine to first simulate and examine the efficacy of the structures before actual fabrication and performance assessment [84-88]. Similarly here at optical frequencies, simulated and measured electromagnetic waves near metallic NPs irradiated by light often agree well; for example, simulation results from nanoscaled antennae which can effectively enhance light emission have been reported [38,39]. As the gap between antennas in microwave engineering and those in optical frequencies is being bridged, nanoantenna structures are promising in further enhancing the LEE in a LED, especially OLED, in which the IQE of the device is already high and cannot be significantly increased. For an LED with a mediocre IQE, nanoantennae can possibly be used to accomplish both IQE and LEE enhancement. Another challenge lies in the fabrication techniques. That is, even though the structures can be designed, they may not be produced using current technology. With regard to the fabrication challenges, efforts are expected to extend existing methods proven useful for arraying ordered metallic arrays such as nanoimprint and chemical synthesis to LED fabrication. The key is to protect the LED structures during chemical processing.

## Competing interests

The authors declare that they have no competing interests.

## Authors' contributions

XG reviewed literatures and drafted the manuscript. TQ and PKC participated in the manuscript drafting and provided constructive opinions in this review paper. WZ also helped to draft the manuscript. All authors read and approved the final manuscript.
